# Supplementary material for: Adaptation of white adipocytes to cooler temperatures: impacts on energy metabolism and protein acetylation
Source: bioRxiv. 2026 Apr 17:2026.04.14.718465. Preprint. [Version 1] doi: 10.64898/2026.04.14.718465 (PMC13104898; doi:10.64898/2026.04.14.718465)
Supplement: Supplement 1 [file media-1.pdf]

Supplementary figures

Adaptation of white adipocytes to cooler temperatures: impacts on energy metabolism and protein acetylation

Hiroyuki Mori[1, #], Hadla Hariri[1, #], William Moe[1], Sophia Durham[1], Yuridia Guzman[1], Emma Paulsson[1], Rachel C. Simmermon[1], Parth B. Bhanderi[1], Sydney K. Peterson[1], Mia J Dickson, Charles R. Evans[2], Ormond A. MacDougald[1,2]

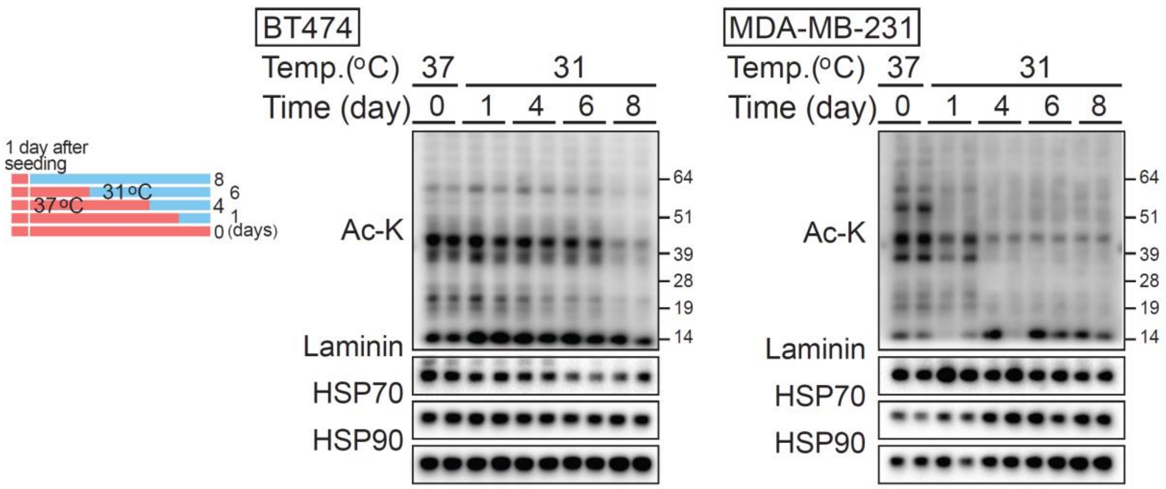

Supplemental Figure 1. The breast cancer cell line BT474 and MDA-MB-231 was incubated at 31°C for the indicated durations. Whole-cell lysates were analyzed by immunoblotting for acetylated lysine, with laminin, HSP70, and HSP90.

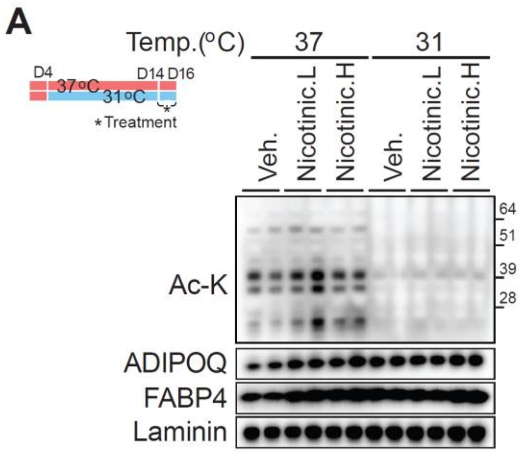

Supplemental Figure 2. Differentiated adipocytes were cultured at either 37°C or 31°C for 10 days, followed by treatment with 20–100 μM nicotinic acid for 2 days.
